# Multi-environment QTL mapping identifies major genetic loci influencing soybean main stem node architecture

**DOI:** 10.7717/peerj.18539

**Published:** 2024-11-26

**Authors:** Honglei Ren, Xue Qu, Huilong Hong, Lingling Sun, Sobhi F. Lamlom, Zhangxiong Liu, Wencheng Lu

**Affiliations:** 1 Soybean Research Institute, Heilongjiang Academy of Agriculture Sciences, Harbin, Heilongjiang, China; 2 College of Modern Agriculture and Ecological Environment of Heilongjiang University, Harbin, Heilongjiang, China; 3 National Key Facility for Crop Gene Resources and Genetic Improvement, Institute of Crop Sciences, Chinese Academy of Agricultural Sciences, Beijing, China; 4 Plant Production Department, Faculty of Agriculture Saba Basha, Alexandria University, Alexandria, Egypt; 5 Heihe Branch Institute, Heilongjiang Academy of Agricultural Sciences, Heihe, China

**Keywords:** Soybean, Main stem node (MSN), QTL mapping, Candidate genes, High-density genetic map, Transcriptional factors

## Abstract

Soybean plant architecture has a significant impact on yield potential, but the genetic underpinnings of key architectural traits remain elusive. The primary objective of this study was to explore the genetic foundations underlying main stem node number (MSN) in soybeans. Recombinant inbred lines (RILs) contained a 234 individual derived from crosses between two cultivars Zhonghuang35 (ZH35) and Jindou21 (JD21) was evaluated for seed hardness across 3 years (2013, 2014, and 2015 in Gansu). Markedly, the parent varieties, shown significant differences in MSN. Also, the RIL population exhibited a wide range of genetic variation in MSN. A high-density genetic map composed of 8,078 specific-locus amplified fragment (SLAF) markers, spanning 3,480.98 centimorgans (cM) with an average inter-marker distance of 0.59 cM were used to construct linkage map. Using ICIM analysis identified a total of 23 Quantitative Trait Loci (QTLs) across the 20 chromosome, of which five QTLs were detected in multiple years in Chr.6. Notably, we identified a stable major QTL, *qMSN-6-4*, explaining up to 24.81% of phenotypic variation. This QTL govern seven candidate genes with potential roles in regulating MSN development in soybean, including *Glyma.06G027500* with a domain of unknown function, *Glyma.06G027600* involved in proton transport, *Glyma.06G027700* linked to proteolysis, *Glyma.06G027900* related to transcriptional regulation, and *Glyma.06G028000* and *Glyma.06G028050* associated with membrane functions. The RT-PCR analysis confirmed that these genes were expressed differently between the parental lines this supports the idea that they may play a role in determining MSN. *Glyma.06G027500* and *Glyma.06G027600* showing higher expression in JD21 leaves and nodes, while *Glyma.06G027700* and *Glyma.06G028000* exhibited increased expression in ZH35 stems, highlighting their distinct roles in transcription regulation, membrane activities, and protein degradation that contribute to MSN formation in soybean. This study offers valuable insights into the genetic mechanisms governing soybean MSN, providing a foundation for future research and crop improvement efforts.

## Introduction

The number of main stem node is the numerical value representing the number of nodes located above the cotyledon node on the main stem (Li et al., 2024). MSN is an ecological characteristic that is influenced by the geographical and sowing seasonal conditions of the growth region (Fahim et al., 2021; Fang et al., 2020). It plays a crucial role in regulating the plant canopy and seed yield potential in soybean. This is because the branches, leaves, pods, and seeds are all arranged on the major stem nodes (Song et al., 2020). Significant correlations were observed between MSN and other crucial agronomic characteristics, including plant height, days to flowering, and days to maturity (Fu et al., 2020). The methodology by which the genetic system of MSN verifies the transmission of this characteristic is highly intriguing to soybean geneticists and breeders (Fu et al., 2017). Nevertheless, MSN is a multifaceted quantitative characteristic regulated by a set of genes and affected by both environmental factors and the specific interplay between genotype and environment (Karikari et al., 2020). These genes effects can vary in magnitude and they can participate in many molecular and biological processes, functioning as a network and exhibiting distinct performance under varying geographical environmental situations (Gai, Wang & Zhang, 2001). Linkage mapping and association mapping are a two generally utilized approaches to identify QTLs architecture of complex traits. Linkage mapping techniques mostly used for single marker analysis (SMA), simple interval mapping (SIM), and composite interval mapping (CIM) (Li & Durbin, 2009). Other methods, such as inclusive composite interval mapping (ICIM) (Li & Durbin, 2009) and mixed model-based composite interval mapping (MCIM), have been developed to estimate the interactions between genotype and environment (G × E) (Lamlom et al., 2024; Lamlom et al., 2020; Yang et al., 2008). However, linkage mapping generally generated mapping findings with the relatively vast region, low marker coverage, high false-positive and false-negative QTL mapping is a highly efficient technique employed to uncover the genetic foundation of fatty acid synthesis (Chen et al., 2007; Ochar et al., 2022). Thus far, several QTLs related to fatty acid content have been identified. Nevertheless, because of the somewhat sparse distribution of genetic maps, these QTLs overlap a significant area of the genome (Fu et al., 2020). The insufficient precision of QTL mapping utilizing these maps has hindered both the discovery of fatty acid biosynthesis and regulatory networks, as well as the use of these QTLs in soybean marker-assisted selection (MAS) breeding endeavors (Chen et al., 2007).

Single nucleotide polymorphisms (SNPs) have become an essential element in genetic map construction, map-based cloning, and genome-wide association analysis due to the release of the soybean reference genome and ongoing advancements in molecular marker technology (Zhang et al., 2013). SNPs can be utilized to identify significant soybean pathways supporting complex quantitative traits (Li et al., 2014). Contemporary researchers have employed specific-locus amplified fragment sequencing (SLAF-seq), the Soybean SNP Bead Chip, SNP genotyping, bin map, and other techniques to ascertain genotypes and generate genetic linkage maps (Jiang et al., 2015). The genetic map markers vary in number from 2,086 to 8,691, collectively covering genetic lengths ranging from 1,478.86 to 3,780.98 centimorgans (cM). The mean map distance ranges from 0.4 to 1.3 cM, and substantial advancements have been achieved in crucial aspects of QTL mapping, including yield (Cao et al., 2017; Liu et al., 2017a; Wang et al., 2016). SLAF-seq is a fast and extensive technique for genotyping single nucleotide polymorphisms (SNPs) using next-generation sequencing (NGS) technology (Li et al., 2014). It achieves efficient read capture and high data processing speed. It finds extensive application in the production of genetic maps, QTL mapping, and other areas of population genetic research associated with molecular breeding (Zhang et al., 2016). Using SLAF-seq technology, several QTLs have been identified in soybean. For instance, a significant QTL related to isoflavone content, named *qIF20-2*, which accounted for 19.6% of the phenotypic variation across various environments (Li et al., 2014). In another study, a recombinant inbred line (RIL) population derived from the cross between ‘Charleston’ and ‘Dongnong 594’ was utilized to identify twelve QTLs associated with oil content; notably, four pairs of epistatic QTLs explained approximately 70% of the phenotypic variation and environmental interactions (Zhaoming et al., 2017). Furthermore, eight out of twenty QTLs associated with phosphorus use efficiency were detected across multiple years and treatments (Li et al., 2017). Nine novel QTLs related to fatty acids were identified, explaining between 0.4% and 37.0% of the phenotypic variations (Li et al., 2017). Most recently, twenty-four stable QTLs for isoflavone components were identified, accounting for 4.2% to 21.2% of the phenotypic variations (Pei et al., 2018). The MSN is influenced by the growth period thus longer growth period in a specific geographic ecoregion and growing season leads to a higher MSN (Fu et al., 2017). Additionally, MSN is linked to attributes of plant structure and productivity potential, including stem growth habit, plant height, branch count, pod count, and seed (Bastidas et al., 2008; Fang et al., 2017). In conjunction with plant height, the MSN calculates the average length of internodes. A study conducted by Fu et al. (2017) demonstrated that in Northeast China, higher-yielding cultivars often exhibit higher MSNs.

Regarding the MSN QTL mapping, of the 13 reported studies, nine were identified utilizing bi-parental populations, whereas five used germplasm populations. On SoyBase (http://www.soybase.org), 38 MSN QTLs by linkage mapping have been collected, whereas in literature, 45 MSN QTLs by GWAS procedures were reported (Assefa et al., 2019; Chang et al., 2018; Fang et al., 2017; Liu et al., 2017b). Recently, 76 MSN QTLs were found, with their genetic contribution approximately 0.04–9.83% per locus for a total of 65.63% for all loci (Assefa et al., 2019; Fu et al., 2020). More recently studies discovered 142 MSN QTLs, from which the evolutionary QTL-allele alterations of MSN among the geographic and seasonal subpopulations of cultivated soybean in the Chinese Soybean Germplasm Population were studied (Wang et al., 2013). Various yield-related QTLs have been discovered in diverse genetic backgrounds and environmental conditions (Tanksley et al., 1988). In the realm of soybean QTL analysis, Keim et al. (1990) were the pioneers in investigating several elements including soybean yield and quality characteristics. Mian et al. (1996) employed two soybean populations to detect QTLs related to seed weight per plant in various conditions and years. They identified two consistent QTLs assigned to linkage groups F and K, respectively. Liu et al. (2011) identified two distinct QTLs associated with seed yield per plant (SYPP) that may be linked to QTLs for seed numbers per pod (SNPP) and pod numbers per plant (PNPP) at the same Chrs 8 locus (Satt390) and 10 locus (Sat_108).

The objective of this investigation is to utilize high-density genetic mapping to identify and characterize significant QTLs that are associated with MSN architecture in soybean. It also aims to identify candidate genes within significant QTL regions, analyze their functions in cellular regulation, and validate their expression patterns through quantitative RT-PCR. Through the investigation of the stability of identified QTLs and the examination of epistatic interactions, the research will offer molecular markers and insights for marker-assisted selection (MAS) in soybean breeding, thereby improving yield stability and architecture.

## Materials and Methods

### Plant materials

The soybean recombinant inbred lines (RILs) originated from the female progenitor, cultivar ‘Zhonghuang 35’ (developed by the Institute of Crop Sciences, Chinese Academy of Agricultural Sciences), and the male progenitor cultivar ‘Jindou 21’ (developed by the Institute of Economic Crops, Shanxi Academy of Agricultural Sciences). The F_2_ population, including 234 individuals, was established in 2008. The F_3_ to F_6_ populations were produced using single seed descent, while the F_6:7_ to F_6:9_ RILs were developed using the multiple seed descent approach. Between 2013 and 2015, the RIL individuals were cultivated at the Dunhuang Experimental Station (N40°17′, E94°65′) of the Gansu Academy of Agricultural Sciences, which receives less than 40 mm of yearly precipitation. The two parents and the RILs were subjected to three replicates of each treatment within a randomized block design, with a row length of 3.0 m, row spacing of 50 cm, plant spacing of 10 cm, and three rows per plot. On April 11^th^ annually, seeds were manually sown, with each hole having two seeds. If both seedlings emerged, one plant was removed following the development of the second trifoliate leaves. The entire field was irrigated at a rate of 120 m^3^ prior to sowing. The field management practices adhered to local production guidelines.

#### Development of the RIL population’s genetic map by simple genome sequencing

##### Development and analysis of SLAF tags

The CTAB method was used to extract genomic DNA from each sample. The construction and sequencing of the SLAF libraries were conducted on 234 RILs and the two parental lines utilizing the SLAF-seq methodology (Biomarker Technologies, Beijing, China) as described by our previous study in 2020 (Ren et al., 2020). We employed *Rsa* I and *Hae* III restriction enzymes to digest genomic DNA in accordance with the soybean reference genome (http://phytozome.jgi.doe.gov/pz/portal.html). We subsequently appended an adenine to the 3′ end of the digested segments and ligated it with the dual index to differentiate the raw sequencing data. We acquired and utilized these fragments to form a sequencing library through PCR amplification, purification, and the combination of PCR products. We conducted sequencing on the Illumina HiSeqTM 2500 platform after obtaining quality certification, which covered sequence amount, base distribution, and sequencing quality distribution. We used rice (*Oryza sativa* L. ssp. japonica cv. Nipponbare) as a control to assess the precision of the SLAF libraries, using the same methodology for library building and sequencing. We grouped the sequences of each sample based on their commonalities and designated them as SLAF markers. The polymorphisms of SLAF markers were discerned by comparing several sample sequences.

### Linkage map construction and QTL identification

A high-density genetic map was constructed using 8,078 out of 43,074 SLAF markers. We assigned markers to 20 chromosomes by aligning them to the reference genome. Linkage groups were designated as chromosomes, and the marker order was determined using HighMap software. The total map length was 3,480.98 cM, with an average inter-marker distance of 0.59 cM, as previously reported by our research team (Ren et al., 2020). Polymorphic SLAF markers were assigned to chromosomes by aligning them with the reference genome. Markers with modified logarithm of odds (MLOD) values below five were excluded from the analysis. Linkage groups were designated as chromosomes, and the marker order was established using HighMap software. Genetic distances between adjacent markers were calculated to construct a high-density genetic map (Meng et al., 2015). QTL mapping for seed hardness was conducted using inclusive composite interval mapping (ICIM) with a step size of 0.1 cM in QTL IciMapping v4.2. Variants located within the identified QTL intervals were selected from the resequencing data, and their effects predicted using SnpEff v5.1. A logarithm of odds (LOD) threshold of 3.9, established based on a 95% confidence interval derived from 1,000 permutations, was applied to identify potential QTL (Zhang et al., 2008).

### Data analysis and QTL mapping

Through the use of the inclusive composite interval mapping (ICIM) approach, we were able to discover MSN QTLs using the QTL IciMapping program (Meng et al., 2015). Based on our estimations from 1,000 permutation tests, we determined the relevance of the QTL declaration. To determine whether a QTL was statistically significant, we utilized a LOD score that was equal to the experimentally determined criterion of *P* = 0.05. Following the idea of QTL naming, we coded all QTL on the same chromosome in the order they were physically located on the chromosome.

### Gene mining and gene annotations in the QTL intervals

Gene mining was conducted using both genetic map positions and physical genome locations to identify candidate genes associated with QTLs related to specific traits. The projection of QTLs from a genetic map onto the soybean reference genome assembly (Glyma.Wm82.a2.v1) was made easier by marker sequences. Additionally screened QTLs that accounted for over 10% of the phenotypic variance and were physically located within 1 Mb for potential underlying candidate genes. We obtained soybean genomic data from the Phytozome website and selected candidate genes based on the physical locations of the major QTLs. The Kyoto Encyclopedia of Genes and Genomes (KEGG) and the Gene Ontology (GO) databases provided comprehensive information regarding pathways, gene ontology, and annotations.

### Quantitative real-time PCR verification

Soybean cultivars JD21 and ZH35 were cultivated in a growth chamber at 26 °C under long-day (16 h light and 8 h dark) conditions. Leaf samples were collected 10 days post-emergence, with each sample comprising material from three individual plants. mRNA extraction was performed using the FastPure Plant Total RNA Isolation Kit (Vazyme, Beijing, China), followed by cDNA synthesis using the HiScript III 1st Strand cDNA Synthesis Kit (Vazyme, Beijing, China). Quantitative PCR (qPCR) was conducted with the ChamQ Universal SYBR qPCR Master Mix (Vazyme, Beijing, China), and three biological replicates were measured for each sample. The qPCR data were analyzed using the 2^−ΔΔCT^ method to determine relative gene expression levels (Livak & Schmittgen, 2001) with GmActin as the internal reference gene (Hu et al., 2009). The specific primers used in the study are detailed in Table 1. To ensure statistical reliability, three independent biological replicates were conducted for each sample.

**Table 1:** Primer information of candidate genes for qRT–PCR.

Gene name	ID	Forward primer	Reverse primer	Temperature	GC%
*Glyma.06G027500*	LOC100805274	CCCACAACACATCCACATAGAAAA	GCCATATCTCGAACCCACCC	59.48	41.67
*Glyma.06G027600*	LOC100805813	AGCAGTGGAGCCTTCAGTTG	CCTGGTACTTGGCGATTCCATA	59.9	55.50
*Glyma.06g027700*	LOC100778716	GAACATGGAGCCGTTACTGGAG	CGCTCTTCCGGATCACACTCA	61.00	54.55
*Glyma.06G027900*	LOC100784080	ACAGATCAACCTGCACAATTCTT	GCTTCTTGTTTGTTGTCCCAAG	58.85	45.45
*Glyma.06g028000*	LOC100786714	TGGCAGTCATACTCAGGATCG	TCTCCCAGTGAGAGTGTTCCA	59.32	52.38
*Glyma.06G028050*	--	AGTTGGAAAGGGTCATGGTGT	GGCCACACACTCTCCACATC	60.13	52.08

## Results

### Variation of soybean main stem node

**Figure 1:**
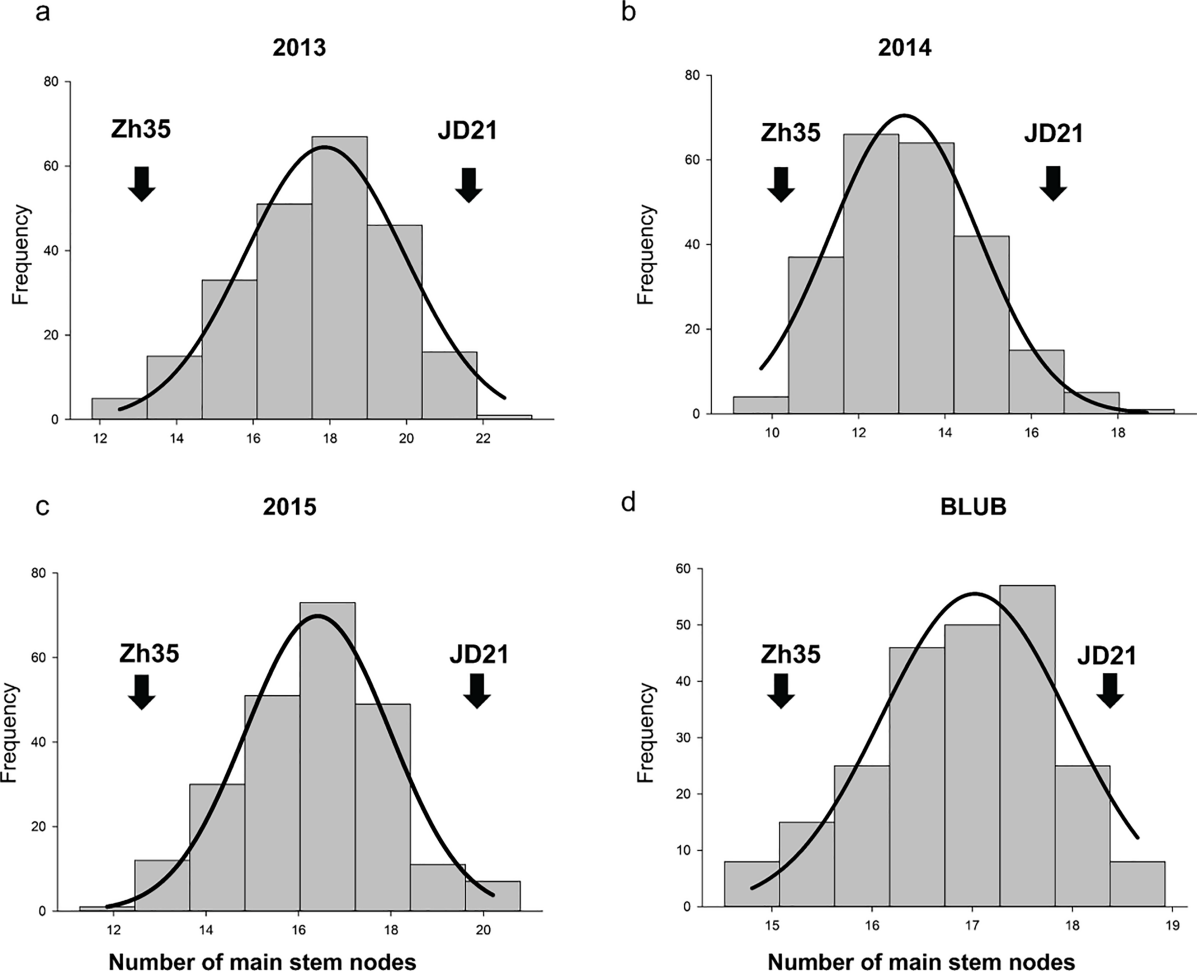
The frequency distribution of the soybean main stem node in the RIL population. (A) Phenotypic variation in 2013. (B) Phenotypic variation in 2014. (C) Phenotypic variation in 2015. (D) Average of phenotypic variation.

In the years 2013, 2014, and 2015, phenotypic analysis of the MSN in soybean showed that the RIL populations had higher mean values than the parent varieties (Table 2, and Fig. 1). Specifically, while the parents Zh35 and JD21 have lower mean values and exhibit greater variation, the RIL populations show more consistency with lower standard deviations. For example, in 2013, the RIL population had a mean of 17.63, which was notably higher than the means of its parent varieties. Over the years, the RIL populations consistently demonstrated higher mean values, indicating a trend towards increased trait expression. The trait distribution in RIL populations is relatively normal, with kurtosis values near zero and skewness values indicating slight deviations from perfect symmetry. These observations suggest that the RIL populations are more stable and consistent in their trait expression compared to their parent varieties. Overall, the data points to effective selection or breeding practices that have enhanced the MSN trait in the RIL populations.

**Table 2:** Phenotypic analysis of main stem node of soybean parents and RIL populations.

	Parent				Population					
Year/Site	Zh35	SD	JD21	SD	Max	Min	Mean	SD	Kurtosis	Skewness
2013	12.19	0.63	17.82	1.22	23.27	11.80	17.63	1.98	−0.36	−0.16
2014	13.77	0.79	17.35	1.86	21.63	11.27	16.34	1.66	−0.06	0.21
2015	12.88	0.72	15.60	1.59	20.80	12.20	16.71	1.57	−0.21	−0.21

### Mapping the QTLs for main stem node

**Figure 2:**
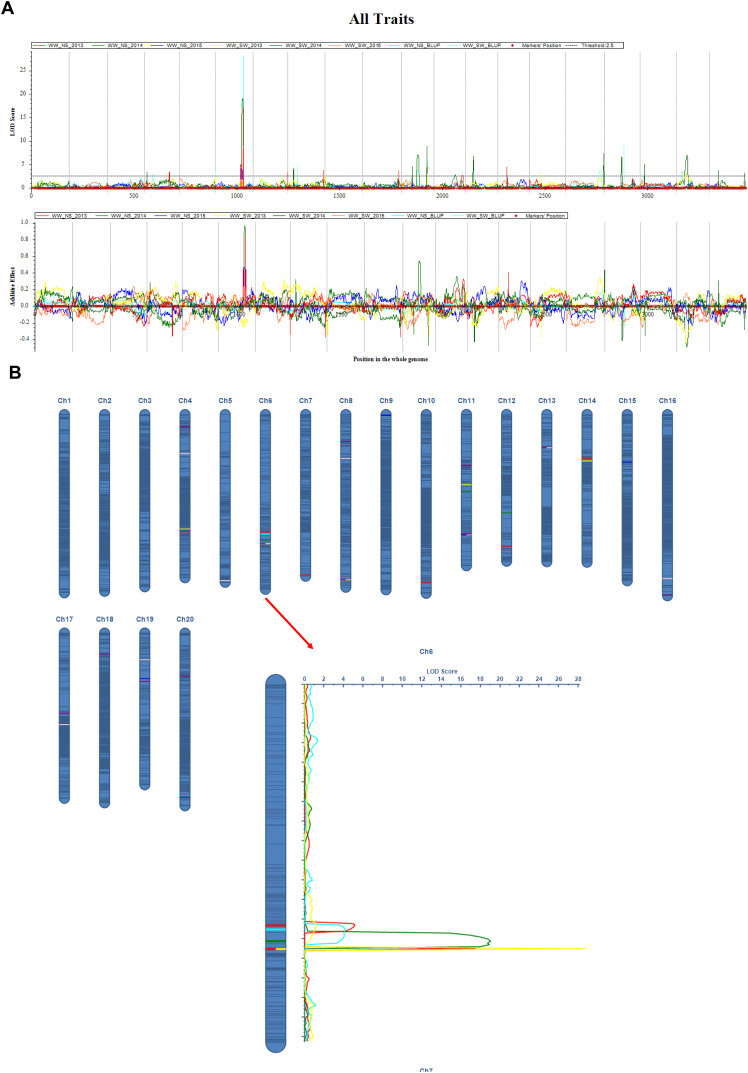
QTL mapping for the soybean main stem node with ICIM-ADD method. (A) LOD curve on the whole genome for MSN; (B) Additive effect on the Ch6 for MSN. The curves indicate the physical position of markers against the LOD score of QTLs detected on chromosomes. Different lines represent different years.

The QTL mapping analysis for the main stem node rate in soybeans shows that this trait is linked to a number of important genetic loci that were found on different chromosomes and in different growing years (Table 3, and Fig. 2). Notably, several QTLs on chromosome 6, including *qMSN-6-3* in 2013 and *qMSN-6-4* in 2014, have high LOD scores and explain a large portion of the phenotypic variation. This suggests that this chromosome is very important for controlling the Main Stem Node rate. In particular, *qMSN-6-3* has a high LOD score of 16.9933 and accounts for 22.51% of the variation, while *qMSN-6-4* explains 24.81% of the variation with a similarly high LOD score. Additionally, *qMSN-7-1* on chromosome 7 stands out with the highest LOD score of 28.1685, explaining 33.82% of the phenotypic variation and highlighting its significant impact on the trait. Other chromosomes, including 8, 10, 11, 12, and 14, also show significant QTLs, though they generally have lower LOD scores and PVE values compared to those on chromosomes 6 and 7. These results emphasize the importance of specific genomic regions in determining the Main Stem Node rate and provide valuable insights for targeted breeding efforts aimed at improving this trait.

**Table 3:** QTL mapping analysis of main stem node rate in soybean.

Year	QTL	Chr.	Position	Left marker	Right marker	LOD	PVE (%)	Add	Genetic interval (bp)
2013	qMSN-4-1	4	123	Marker1731877	Marker1744144	3.43	4.18	−0.36	40,917,597–41,585,415
BLUP	qMSN-6-1	6	120	Marker1672203	Marker1722576	3.25	3.033	−0.16	39,268,439–39,493,499
2013	qMSN-6-2	6	123	Marker2170727	Marker2169539	5.12	6.38	0.45	16,417,845–37,072,547
2013	qMSN-6-3	6	135	Marker2150823	Marker2121401	16.99	22.50	0.85	35,541,410–38,332,813
2014	qMSN-6-4	6	131	Marker2169539	Marker2178329	19.04	24.80	0.96	37,072,230–19,480,263
2015	qMSN-6-5	6	125	Marker2170727	Marker2169539	4.14	7.60	0.47	16,417,845–37,072,547
BLUP	qMSN-7-1	7	135	Marker2150823	Marker2121401	28.16	33.81	0.56	35,541,410–38,332,813
2013	qMSN-8-1	8	169	Marker1548466	Marker1514921	3.23	3.79	−0.34	43,196,008–43,383,184
2013	qMSN-8-2	8	173	Marker1086673	Marker1048097	3.74	4.36	−0.37	43,057,788–43,335,641
BLUP	qMSN-10-1	10	173	Marker1086673	Marker1048097	3.28	3.01	−0.16	43,057,788–43,335,641
2013	qMSN-11-1	11	175	Marker656413	Marker631714	3.72	4.30	−0.36	2,666,511–2,562,259
2014	qMSN-11-2	11	81	Marker528494	Marker517683	7.05	8.06	0.53	15,208,131–11,274,577
BLUP	qMSN-12-1	12	74	Marker516098	Marker562768	3.78	3.47	0.17	15,599,858–15,813,263
2013	qMSN-12-2	12	139	Marker1866106	Marker1818174	2.61	3.17	0.31	4,284,454–2,784,376
2014	qMSN-14-1	14	104	Marker1803106	Marker1816956	2.7081	3.46	0.35	8,929,281–12,161,672
2013	qMSN-14-2	14	46	Marker1609150	Marker1652910	4.4084	5.13	0.40	28,017,416–27,668,792

### Candidate gene mining and expression analysis

In our study, we conducted a comprehensive candidate gene mining and expression analysis to elucidate the genetic underpinnings of the MSN trait in soybean, a critical determinant of plant architecture and yield potential as shown in Table 4. By focusing on a stable QTL, *qMSN-6*, identified across multiple environments, we performed detailed GO and candidate gene prediction analyses within the QTL’s physical genomic region. Our investigation identified several candidate genes with putative roles in cellular and molecular processes relevant to MSN development. Among the genes identified, *Glyma.06G027500* encodes a protein with a domain of unknown function (DUF702), suggesting a potentially novel role in cellular regulation. *Glyma.06G027600* was annotated as part of the V-type ATPase 116kDa subunit family, implicated in proton transport and energy metabolism within the plasma membrane and endomembrane systems. Similarly, *Glyma.06G027700* shows homology to a cysteine protease family protein, highlighting its possible involvement in proteolytic processes that regulate cellular homeostasis. The gene *Glyma.06G027900* aligns with a transcription initiation factor subunit, underscoring a likely role in the transcriptional regulation of genes linked to growth and development. Finally, *Glyma.06G028000*, and *Glyma.06G028050* were found to be associated with membrane-related functions, including plasma membrane structure and peptide transport, respectively.

**Table 4:** Functional annotations of candidate genes associated with main stem node development in soybean.

Gene	KEGG annotation information	GO annotation information	Pfam annotation information	Homologous gene	Homologous gene description
*Glyma.06G027500*		GO:0005634	PF05142	AT3G51060.1	Domain of unknown function (DUF702) (DUF702)
*Glyma.06G027600*	K02154	GO:0012505 GO:0005886	PF01496	AT4G39080.1	V-type ATPase 116 kDa subunit family
*Glyma.06G027700*	K01373	GO:0005615	PF08246	AT4G39090.1	Cysteine protease family c1-related
*Glyma.06G027800*		GO:0012505 GO:0031090			Unknown
*Glyma.06G027900*	K03139	GO:0005634	PF02270	AT3G52270.1	Transcription initiation factor TFIIF subunit beta (TFIIF2, GTF2F2, TFG2)
*Glyma.06G028000*		GO:0005886 GO:0012505	PF00892	AT1G75500.1	Protein walls are thin 1
*Glyma.06G028050*		GO:0005615			Peptide transporter

### Analysis of candidate gene expression

**Figure 3:**
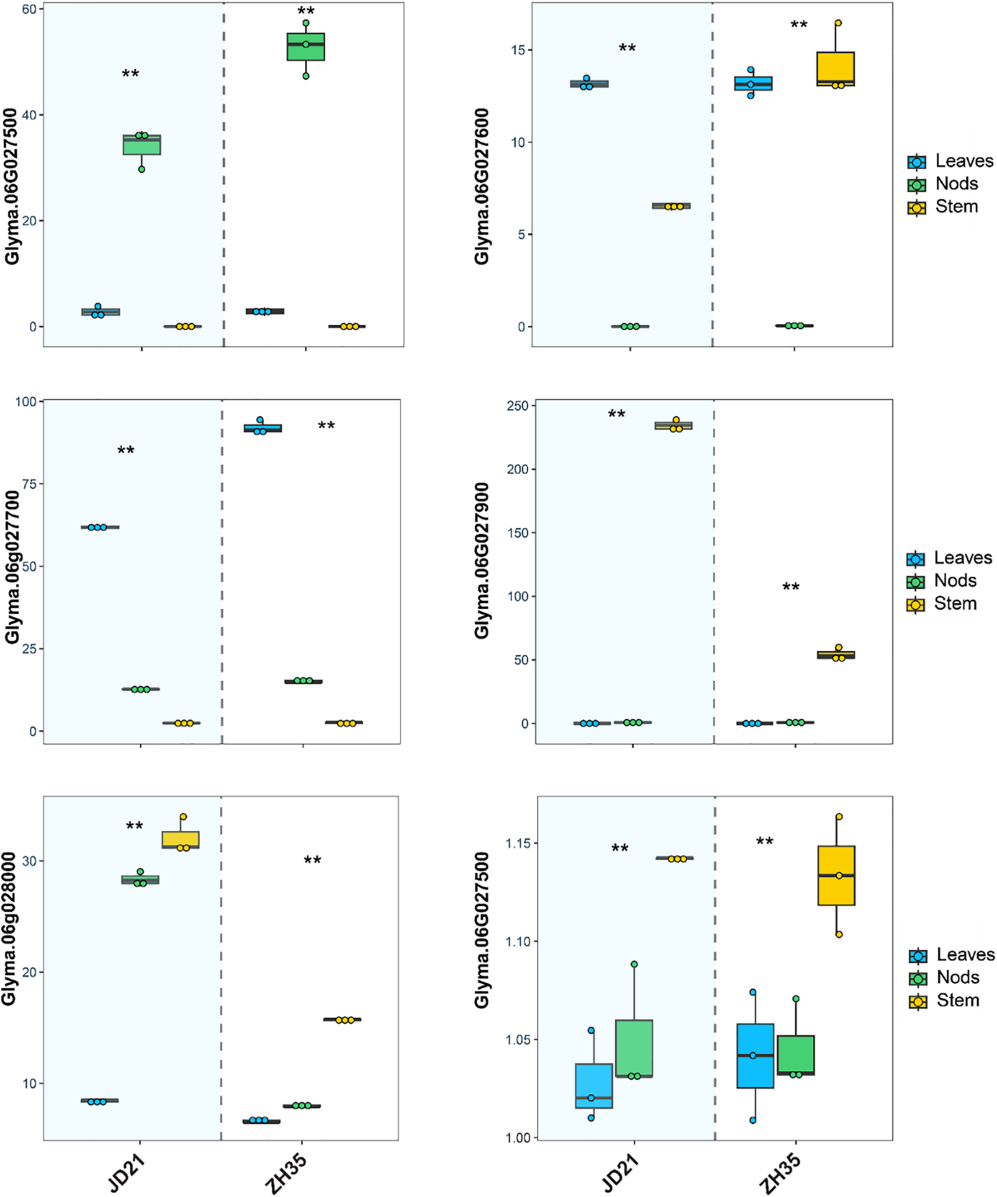
Relative expression levels of *six candidate gene* in JD21 and ZH35. ^**^Indicated expression level was significantly different at *P* < 0.05.

The RNA expression investigation of six candidate genes linked to main stem node development in soybean revealed tissue-specific and condition-dependent differences in gene expression across the JD21 and ZX35 parents’ leaves, nodes, and stems (Fig. 3). Specifically, *Glyma.06G027500*, which has a domain of unknown function (DUF702), showed significantly higher expression in the leaves and nodes of the JD21 cultivar compared to the ZH35 cultivar, which had significantly lower expression across all tissues, especially in the stem. This indicates that *Glyma.06G027500* may have a more significant involvement in cellular processes occurring in the aerial regions of the plant, especially in the leaves and nodes of JD21. *Glyma.06G027600*, which is linked to V-type ATPase complex functionality, showed significantly elevated expression in JD21 nodes relative to ZH35, as well as a marked reduction in stem expression in ZH35. This pattern emphasizes its essential role in nodal development, possibly linked to energy metabolism and intracellular transport, which may be particularly significant in the JD21 cultivar. *Glyma.06G027700*, a gene belonging to the cysteine protease family, had modest expression in all tissues of JD21 but showed a significant increase, particularly in ZH35 stem tissues. The upregulation suggests that ZH35 promotes conditions that increase gene expression, possibly associated with protein breakdown or remodeling processes in growth signals inside the stem. *Glyma.06G027800* demonstrated a consistently steady expression profile across many tissues and circumstances, with a slight elevation observed in the ZH35 stem. The consistent expression suggests that *Glyma.06G027800* probably has a general maintenance role, possibly connected with the endomembrane system, rather than being specifically responsive to changes in development or the environment. Both cultivars showed elevated expression of *Glyma.06G027900*, associated with transcription initiation (TFIIF subunit beta), in nodes; however, ZH35 showed a minor decrease. The gene’s stable expression in nodes during both treatments suggests that it continues to play a role in controlling transcription during node development. This may include controlling the expression of other genes that are necessary for stem node formation. *Glyma.06G028000*, which is linked to activities in the plasma membrane, showed a big rise in stem expression in cultivar ZH35 compared to JD21. The higher level of expression in the stem suggests that this gene may be involved in activities or signaling pathways that are connected to membranes and are important for the development of structure in stem tissues. Synopsis The RNA expression profiles of the six candidate genes exhibit distinct patterns between the JD21 and ZH35 cultivars, with some genes demonstrating elevated expression in JD21, particularly in nodes and leaves, while others, such as *Glyma.06G027700* and *Glyma.06G028000*, show heightened expression in ZH35, especially in stems. The tissue- and condition-specific expression alterations indicate that these genes participate in several biological processes, including transcription control, membrane activities, and protein degradation, which are essential for stem node formation and possibly for soybean.

## Discussion

The utilization of QTL mapping has proven to be a very efficient approach for the analysis of several traits in plants. The main factors influencing the efficiency and accuracy of QTL mapping are marker density and parental genetic diversity. The methodology of QTL mapping is extraordinarily valuable for the examination of quantitative traits in plants (Ahmed et al., 2024; Kumar et al., 2023; Liu et al., 2023). Nevertheless, the accuracy of QTL mapping is heavily influenced by the quality of genome maps. The resolution of genetic maps can be improved by increasing the density of genetic markers, as demonstrated by studies conducted by Guo et al. (2005), Hirata et al. (2014), McCouch et al. (1997). However, soybean demonstrates far greater levels of linkage disequilibrium (LD) in comparison to other plants (Ochar et al., 2022; Orazaly et al., 2018). SLAF-seq is a proficient sequencing technique for extensive marker identification and genotyping, utilized for genetic study across various species (Guo et al., 2015; Ji et al., 2017; Jiang et al., 2015; Li et al., 2016; Liu et al., 2014; Zhang et al., 2013). In our previous research, we employed SLAF-seq to discover markers extensively and genotype individual samples, resulting in a highly detailed genetic map (Ren et al., 2023; Zhang et al., 2023). SLAF-seq has numerous advantages, including efficient marker selection, cost-effectiveness, and the ability to examine large populations. SLAF-seq technology produced a detailed genomic map of soybean, comprising 8,078 markers. The cumulative genetic distance among these markers was 3,780.98 cM, with an average interval of 0.59 cM. This map has markedly improved marker density and reduced inter-marker distance compared to previous soybean genetic maps. A close study of the high-density genetic map made it easier to find quantitative trait loci (QTLs) that were connected to plant height, seed weight per plant, and drought tolerance coefficient in several different ecological settings. The MSN features of the RIL population originating from ZH35 and JD21 exhibited continuous distributions, either normal or skew normal. Enhancing marker density may improve the genetic map’s resolution for a specific mapping population. To make this study’s high-density map, we used 5,111 high-quality SLAF markers and then put together 8,597 SNP loci into 20 linkage groups. This high-density genetic map enhances the accuracy and reliability of QTL mapping, hence benefiting MAS breeding.

MSN of soybean plants is a crucial factor in determining the architecture of the plants, namely the number of branches, which ultimately impacts the seed output. As the management of axillary bud extension after axillary meristem initiation is governed by intricate spatial-temporal regulation, identifying the genetic foundation has significant potential to improve the selection and effectiveness of breeding for high-yielding soybean cultivars. This work identified a total of 23 QTLs for MSN using a high-density map generated from an F_6:11_ RIL population consisting of 234 individuals resulting from the cross between ZH35 and JD21. Our results provide valuable understanding of the genetic and phenotypic diversity of the MSN characteristic in soybean, which has substantial consequences for plant structure and crop productivity. The phenotypic data analysis conducted over three years (2013, 2014, and 2015) reveals a persistent rise in the average values of MSN in the recombinant inbred line (RIL) populations when compared to the parent varieties, Zh35 and JD21. The observed trend, along with the reduced standard deviations and reasonably normal distribution of traits in the RIL populations, implies a more consistent expression of MSN. This demonstrates that the breeding methods used were successful in improving this characteristic. The increased average MSN levels seen in the RIL populations may be ascribed to the progressive accumulation of advantageous alleles from both parent types, hence augmenting the overall expression of the trait.

The QTL mapping analysis further supports these phenotypic observations by identifying multiple quantitative trait loci (QTLs) associated with the MSN trait. Notably, chromosomes 6 and 7 harbor key QTLs (*qMSN-6-3*, *qMSN-6-4*, and *qMSN-7-1*) with high logarithm of odds (LOD) scores and substantial phenotypic variation explained (PVE). The identification of *qMSN-7-1* on chromosome 7, which accounts for 33.82% of the phenotypic variation and exhibits the highest LOD score, highlights its major role in controlling MSN. Additionally, the detection of several QTLs on chromosome 6 suggests the involvement of multiple genomic regions within this chromosome in the regulation of MSN. The clustering of QTLs on specific chromosomes, coupled with their high PVE values, indicates that these loci are key contributors to the expression of the MSN trait and could serve as valuable targets for marker-assisted selection in breeding programs.

Linked analysis revealed 25 QTL and Genome-Wide Association Studies (GWAS) detected in SoyBase (https://legacy.soybase.org/search/qtllist.php) (Li et al., 2021). Out of the 119 QTL discovered in our work, 10 had genomic intervals that coincided with the QTL node number reported in public literature. qlNN-2-1 was discovered on chromosome 2 within the genomic intervals of 29,959,409–41,608,316 base pairs (bp), which coincide with node number 4-1 (38,221,027–40,699,300 bp) as reported by Liu et al. (2011). The qlNN-5-1 gene was found on chromosome 5 at the genomic intervals of 22,088,622–41,360,809 base pairs (bp), which coincides with node number 3-1 (35,971,621–38,939,759 bp) as reported by Chen et al. (2007). The qlNN-6-2 segment was found on chromosome 6 within the genomic intervals of 11,860,267–12,150,538 base pairs (bp), which coincides with node number 5-1 (10,251,126–12,336,492 bp) as reported by Moongkanna et al. (2011). The qlNN-13-1 (qlRDNN-13-1) gene was found on chromosome 13 at the genomic intervals of 444,838–43,052,819 base pairs. It overlaps with node numbers 1–5, 1–6, 1–7, 1–8 as reported by Gai et al. (2007), and 2-3 as reported by Zhang et al. (2004). The qlNN-17-2 gene was found on chromosome 17 at the genomic intervals of 7,296,590−9,660,500 base pairs (bp), which coincides with node number 7-1 (5,788,551–9,576,644 bp) as reported by Li et al. (2020). The genes qnNN-5-4 (37,951,491 bp) and qnRDNN-5-4 (38,349,709 bp) were found on chromosome 5 throughout the range of node number 3-1 (35,971,621–38,939,759 bp) as reported by Chen et al. (2007). Similarly, qnNN-6-3 (19,386,897 bp) was found on chromosome 6 within the range of node number 2-2 (19,370,872-20,218,893 bp) as reported by Zhang et al. (2004). DNA fragments qnRDNN-13-1 (12,074,020 base pairs) and qnNN-13-1 (14,139,382 base pairs) were located on chromosome 13 and were found within the range of node number 1-5 (10,199,530–15,306,234 base pairs) (Gai et al., 2007).

The identification of stable QTLs across different years and environments, such as *qMSN-6-1*, underscores their potential use in developing soybean varieties with optimized MSN traits. The consistency of these QTLs across diverse environments suggests that they may confer resilience to varying growing conditions, further enhancing their value in breeding programs. The candidate gene mining within the QTL regions provides deeper insights into the molecular mechanisms underlying MSN development. For instance, the discovery of *Glyma.06G027500*, which encodes a protein with a domain of unknown function (DUF702), points to the presence of potentially novel regulatory pathways influencing this trait. Similarly, genes like *Glyma.06G027600* and *Glyma.06G027700*, involved in proton transport, energy metabolism, and proteolytic processes, respectively, suggest that a complex network of cellular processes contributes to MSN expression. The identification of genes such as *Glyma.06G027900*, which aligns with a transcription initiation factor subunit, further highlights the role of transcriptional regulation in controlling growth and development traits like MSN. The involvement of genes related to membrane functions and peptide transport, such as *Glyma.06G028000* and *Glyma.06G028050*, suggests that the regulation of MSN may also be linked to membrane dynamics and transport processes, which are critical for cell signaling and development.

## Conclusion

The extensive investigation of the genetic structure that governs the formation of main stem nodes (MSN) in soybean has resulted in several noteworthy findings. By employing high-density genetic mapping and multi-environment phenotyping, we have successfully discovered several quantitative trait loci (QTLs) that regulate MSN, a crucial factor influencing the structure and productivity of soybean plants. The identification of a prominent stable quantitative trait locus (QTL), *qMSN-6-4*, which accounts for up to 24.81% of phenotypic variance, offers a substantial progress in our comprehension of the genetic regulation of this crucial characteristic. This study establishes a basis for further functional characterisation by identifying six potential genes inside the *qMSN-6-1* interval that are believed to be involved in cellular regulation, energy metabolism, and transcriptional control. The variability in the expression of these genes among parental lines, as verified by qRT-PCR analysis, provides evidence for their possible role in determining MSN. These findings deepen the understanding of plant architectural development and offer valuable targets for marker-assisted selection and molecular breeding. The research illustrates the efficacy of combining high-throughput genotyping technologies, multi-environment phenotyping, and functional genomics techniques for analyzing complex agronomic traits.

### Future aspects

The utilization of this integrative approach can function as a paradigm for exploring additional significant characteristics in soybean and other crop species, thereby potentially expediting the rate of crop enhancement. The clarification of the genetic foundation of MSN presents novel opportunities for refining soybean plant structure to maximize light absorption, enrich photosynthetic efficiency, and eventually raise crop productivity. Moreover, the identification of certain genetic loci and candidate genes serves as a foundation for investigating the molecular processes that govern the interaction between plant structure and environmental adaptation. Future research should prioritize the validation of the functions of the candidate genes that have been found using functional genomics methodologies, such as gene editing or transgenic experiments. Furthermore, it is essential to study the possible pleiotropic impacts of the discovered QTLs on other agronomically significant characteristics in order to promote comprehensive breeding approaches. Ultimately, investigating the preservation of these genetic processes among many legume species could offer more comprehensive understanding of the development of plant structure in this significant plant group. In summary, our work represents a substantial advancement in comprehending the genetic regulation of soybean plant structure. The results of this study have direct practical implications in molecular breeding initiatives and establish the foundation for further explorations into the underlying biology of plant structure and function.

## Supplemental Information

10.7717/peerj.18539/supp-1 Supplemental Information 1 Raw data.

10.7717/peerj.18539/supp-2 Supplemental Information 2 RT-qPCR.

10.7717/peerj.18539/supp-3 Supplemental Information 3 MIQE Checklist.

## Additional Information and Declarations

### Data Availability

The following information was supplied regarding data availability: The raw data is available in the Supplemental File.
